# Safety and efficacy of administering reduced doses of pegylated recombinant human granulocyte‐colony stimulating factors in patients treated with cisplatin and etoposide for small cell carcinoma: A retrospective study

**DOI:** 10.1111/1759-7714.13883

**Published:** 2021-02-15

**Authors:** Chang Liu, Ying Hao, Lei Wang, Fanlu Meng, Fuyu Wen, Diansheng Zhong

**Affiliations:** ^1^ Tianjin Medical University General Hospital Tianjin China; ^2^ Tianjin Chest Hospital Tianjin China

**Keywords:** febrile neutropenia, etoposide and cisplatin, neutropenia, PEG‐rhG‐CSF

## Abstract

**Background:**

The aim of this study was to discuss the safety and efficacy of administering reduced doses (3 mg) of pegylated recombinant human granulocyte‐colony stimulating factor (PEG‐rhG‐CSF) at approximately 24 h or up to three days following treatment with etoposide and cisplatin (EP).

**Methods:**

A total of 104 cycles from 31 patients were divided into a PEG‐rhG‐CSF prophylaxis group (PP‐Group) and a control group (No‐PP‐Group). The PP‐Group received a reduced dose of 3 mg of PEG‐rhG‐CSF within a minimum of 15 h and a maximum of 72 h following EP chemotherapy, while the rest did not receive any G‐CSF prophylaxis (No‐PP‐Group). For both groups, complete blood counts, incidence of febrile neutropenia (FN), grade III or IV neutropenia, and the use of antibiotics to treat neutropenia were recorded.

**Results:**

There was statistically no significant difference in the incidence of FN (0% vs. 1.4%, *p* = 1), antibiotic use due to neutropenia (0% vs. 2.7%, *p* = 0.881), estimated lowest mean marginal (EM) platelet (106.56 × 10^9^/L vs. 127.70 × 10^9^/L, *p* = 0.056) and hemoglobin (110.48 g/L vs. 110.14 g/L, *p* = 0.906) levels between the two groups. However, when compared with the No‐PP‐group, the white blood cell count in the PP‐group was significantly higher (EM means: 4.95 × 10^9^/L vs. 2.80 × 10^9^/L, *p* < 0.01), while the incidence of grade III or IV neutropenia was significantly lower (9.1% vs. 68.1%, *p* < 0.01).

**Conclusions:**

The administration of a low dose (3 mg) of PEG‐rhG‐CSF within approximately 24 h or up to three days following EP treatment is safe and effective at reducing the risk of neutropenia. These findings bring a more flexible administration interval between PEG‐rhG‐CSF and EP treatment.

## Graphical abstract

Even though cisplatin is slowly metabolized by the body, the routine administration time of PEG‐rhG‐CSF following cisplatin was safe. A reduced dose (3 mg) of PEG‐rhG‐CSF was also effective in preventing grade III and IV neutropenia.

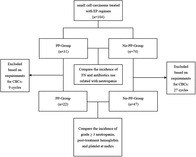

## INTRODUCTION

Patients diagnosed with small cell carcinoma and treated with cytotoxic chemotherapy using etoposide and cisplatin (EP) are at intermediate risk of developing myelosuppression and febrile neutropenia (FN), potentially leading to the development of life‐threatening infections. Granulocyte colony‐stimulating factors (G‐CSFs) are growth factors that can stimulate the bone marrow to produce neutrophils and minimize the risk of developing FN in these patients. Pegylated recombinant human granulocyte‐colony stimulating factor (PEG‐rhG‐CSF) is currently the most commonly used prophylactic G‐CSF agent in clinical practice.^1^, ^2^, ^3^ Improved patient compliance with chemotherapy treatment further favors its use clinically.^4^

According to the National Comprehensive Cancer Network (NCCN),^11^ G‐CSF should be administered 24 to 96 h following the cisplatin infusion. However, the elimination half‐life of cisplatin from the circulation is about 48 h, much longer than that of etoposide, which is about 7 h.^9^, ^10^ Within 24 h following the administration of cisplatin, only about 9.52% to 26.9% of the platinum is excreted in the urine,^18^ while about 27% to 45% is excreted within the first five days of treatment.^19^ If PEG‐rhG‐CSF is administered only 24 h after EP treatment, cisplatin will still be present in the blood. This could potentially compromise the production of myeloid progenitor cells induced by PEG‐rhG‐CSF,^5^ eventually rendering the treatment ineffective.^2^, ^6^, ^7^, ^8^ On the other hand, other studies argue that when cisplatin is infused into the blood, it binds rapidly to plasma proteins.^20^ The bound fraction increases with time and can reach 90% within 2 h after the end of infusion.^20^ It is known that only the unbound drug has therapeutic and toxic activities.^10^ However, the free platinum is rapidly cleared from plasma with an elimination half‐life of only about 22 min.^10^ All the above factors can guarantee that the concentration of the free platinum will be at low levels within the plasma when PEG‐rhG‐CSF is applied even within 24 h after the cisplatin. This means that PEG‐rhG‐CSF treatment might be safely administered on the same day with the cisplatin, making it more convenient as the patient does not need to return to the hospital for treatment.

Consequently, more research is required to identify the safety of various intervals between the PEG‐rhG‐CSF treatment and the EP regimen, and the impact of this treatment on platelet, hemoglobin and white blood cell (WBC) levels.

Furthermore, the standard recommended dose of PEG‐rhG‐CSF is currently 6 mg. However, some patients may not be able to afford this treatment, leading to poor compliance. Evidence indicates that the early discontinuation of PEG‐rhG‐CSF is related to a higher risk of developing FN in subsequent chemotherapy cycles.^12^ Conversely, some small retrospective scale studies have shown that the administration of half the dosage of PEG‐rhG‐CSF might still achieve a prophylactic effect, eventually providing these patients with a safe and more cost‐effective alternative.^13^, ^14^ In view of this, the use of half the standard dose (3 mg) of PEG‐rhG‐CSF has been tried as an alternative prophylactic treatment in China.

Therefore, the study aimed to retrospectively evaluate the safety and efficacy of administering a 3 mg dose of PEG‐rhG‐CSF at approximately 24 h or up to three days following the EP treatment in reducing the incidence of neutropenia in patients diagnosed with small cell carcinoma.

## METHODS

Patients diagnosed with small cell carcinoma between April 11, 2019 to April 11, 2020 and followed up to May 4, 2020 at the General Hospital of Tianjin Medical University were identified. The complete blood counts (CBCs) of each patient were obtained from their medical records, while the “WeChat” follow‐up group was used to obtain information about the patients' condition post‐treatment. All patients older than 18 years who had a biopsy‐confirmed diagnosis of small cell carcinoma were included in the study. All patients received one to six cycles of EP. The cisplatin dosage ranged from 80 to 100 mg/m^2^, either administered in one day or distributed over three days, and was combined with an etoposide dose ranging from 75 to 80 mg/m^2^ on day one through three. Patients who had their chemotherapy dosages reduced due to high levels of treatment‐related toxicity were also included.

### PEG‐rhG‐CSF prophylaxis

**FIGURE 1:**
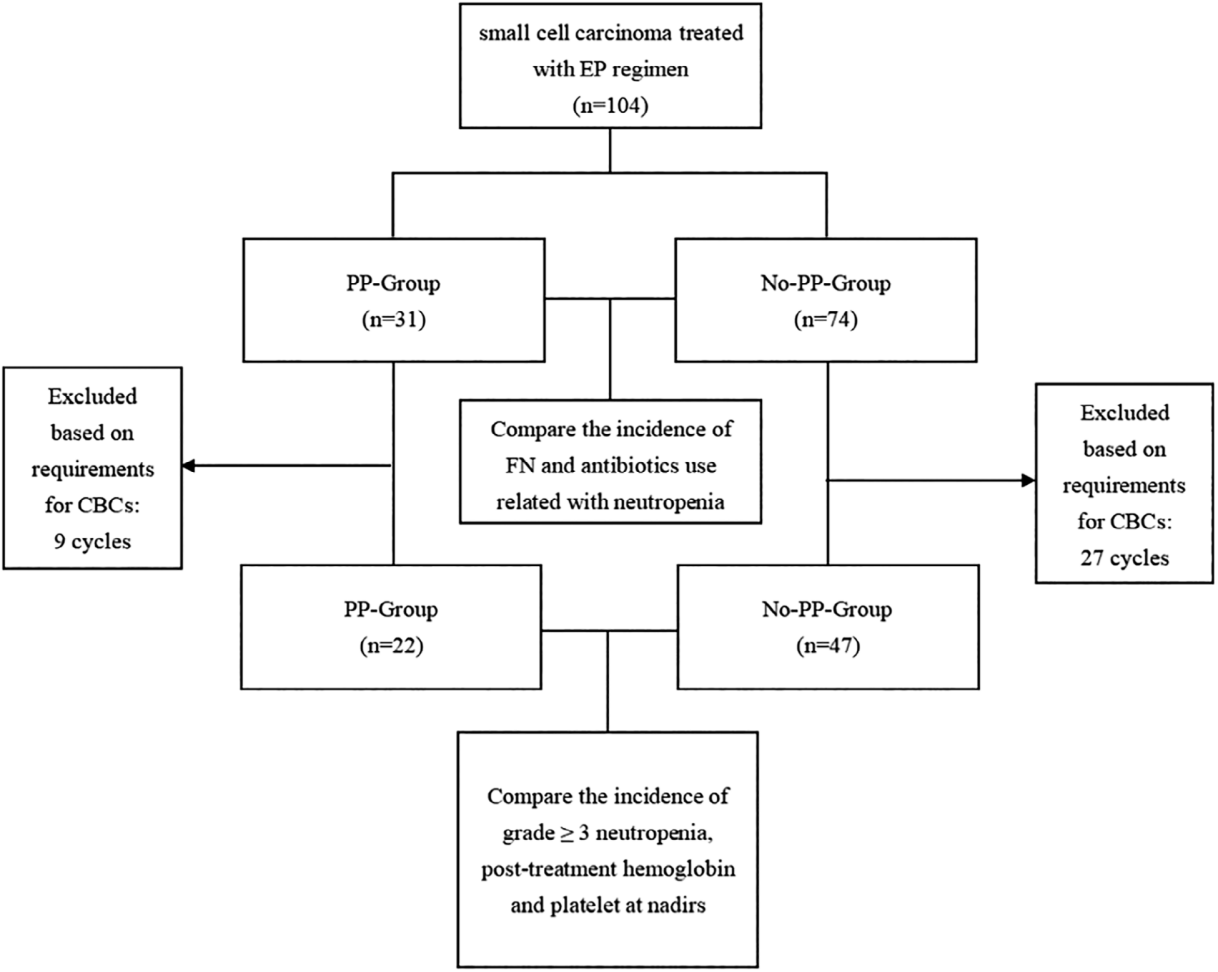
Procedures of the study. EP, etoposide, and cisplatin; PP, PEG‐rhG‐CSF prophylaxis; FN, febrile neutropenia; CBCs, complete blood counts

A total of 104 cycles from 31 patients were divided into a PEG‐rhG‐CSF prophylaxis group (PP‐Group) and a control group (No‐PP‐Group) (Figure 1). The PP‐Group received a reduced dose (3 mg) of PEG‐rhG‐CSF within a minimum of 15 h and a maximum of 72 h after the last dose of chemotherapy infusion. All patients in the PP‐Group received PEG‐rhG‐CSF as a secondary prophylactic use after experiencing grade III or IV neutropenia in previous cycles or if their previous chemotherapy treatment had to be delayed due to neutropenia. The No‐PP‐Group did not receive any prophylactic use of PEG‐rhG‐CSF.

### Data analysis

The incidences of FN and antibiotic use due to neutropenia were ascertained after each cycle. FN was defined as either an oral temperature higher than 38.0°C or an axillary temperature higher than 38.1°C or two consecutive axillary temperature readings above 37.8°C acquired within 2 h and an absolute neutrophil count (ANC) below 0.5 × 10^9^/L, or an ANC expected to fall below this level.^15^, ^16^

The patient data were retained for further analysis if the patient had a CBC between the seventh day and the fifteenth day of the chemotherapy cycle, or at least once within 5 to 7 days after the administration of PEG‐rhG‐CSF,^17^ or if the patients experienced grade three or four neutropenia with an ANC below 1.0 × 10^9^/L at any point during the chemotherapy treatment and at least one test from the sixteenth dayuntil the next chemotherapy cycle (Figure 1).

Descriptive statistics were used to quantify the clinical characteristics of patients, including sex, age, bodyweight, cancer site, days of cisplatin usage, current chemotherapy cycle, chemotherapy purpose, history of radiation and range of pretreatment ANC for each chemotherapy cycle. The Chi‐square test (continuity correction for the expected count of any cell less than five) was used to compare the incidences rates of FN and antibiotic use due to neutropenia, while the Pearson's chi‐square test was used to compare the rates of grade III or IV neutropenia between the two groups. Covariance analysis was used to evaluate the impact of administering PEG‐rhG‐CSF on the CBC. The average estimated marginal (EM) platelet, hemoglobin and WBC counts post‐treatment were also calculated. The average EM represented the expected mean for each variable after adjusting for covariables. All statistical tests were performed using the Statistical Package for Social Sciences (SPSS) software, version 25.0, and a *p*‐value below 0.05 was deemed to be statistically significant.

### Study endpoints

The primary study safety endpoints were defined as the number of chemotherapy cycles with FN, antibiotic use due to neutropenia, and the post‐treatment platelet and hemoglobin counts. The secondary efficacy endpoint was defined as the efficacy of a 3 mg PEG‐rhG‐CSF dose in reducing the incidences of grade III and IV neutropenia.

### Ethical considerations

All procedures performed in this study involving human participants were performed in accordance with the ethical standards of Tianjin Medical University General Hospital and with the 1964 Helsinki declaration and all subsequent revisions. Since this study was retrospective, obtaining informed consent from each patient was not required.

## RESULTS

Thirty‐one patients fulfilled the inclusion criteria, and a total of 104 chemotherapy cycles were evaluated. Prophylactic PEG‐rhG‐CSF use was provided for 31 of the chemotherapy cycles, while no PEG‐rhG‐CSF was provided for the rest of the cycles.

### Incidences of FN and antibiotic use due to neutropenia

There was no statistically significant difference in the incidence of FN (0% vs. 1.4%, *p* = 1) between the PP‐Group and No‐PP‐Group, respectively. One case in the No‐PP‐Group showed grade IV neutropenia accompanied by infectious signs with no fever, which was treated with antibiotics. There was no statistically significant difference in the incidence of antibiotic use due to neutropenia in the PP‐Group and No‐PP‐Group (0% vs. 2.7% *p* = 0.881) (Table 1). No mortality associated with neutropenia or infection occurred in any of the groups.

**TABLE 1:** Incidences of FN and antibiotic use due to neutropenia from any cycle of chemotherapy

	PP‐Group (*n* = 31)	No‐PP‐Group (*n* = 73)	*p*‑value
FN	0 (0%)	1 (1.4%)	1
Antibiotic use due to neutropenia	0 (0%)	2 (2.7%)	0.881

Abbreviations: FN, febrile neutropenia; PP, PEG‐rhG‐CSF prophylaxis.

### Clinical characteristics of remaining data for further analysis

After filtering out the data that did not meet the requirements for further analysis, a total of 69 cycles from 26 patients were left; 22 from the PP‐Group, and 47 from the No‐PP‐Group. The clinical characteristics of the cycles are further summarized in Table 2. The composition of “current chemotherapy cycle” varied significantly between the two groups. None of the patients received PEG‐rhG‐CSF prophylaxis in the first chemotherapy cycle. However, after the first two chemotherapy cycles, many patients required prophylactic use of PEG‐rhG‐CSF, eventually resulting in a larger number of patients starting with their third chemotherapy cycle in the PP‐Group. For all other clinical and treatment characteristics, the difference between the two groups was not statistically significant.

**TABLE 2:** Clinical characteristics of patients having regular CBCs

Clinical characteristics	PP‐Group (*n* = 22)	No‐PP‐Group (*n* = 47)	*p*‑value
Sex			0.336
Male n (%)	17 (77.3)	42 (89.4)	
Female n (%)	5 (22.7)	5 (10.6)	
Age (years)	64.59 ± 5.92	61.83 ± 6.70	0.103
Bodyweight (kg)	69.32 ± 10.64	68.07 ± 10.49	0.649
<60 kg n (%)	2 (9.1)	8 (17.0)	0.613
≥60 kg n (%)	20 (90.9)	39 (83.0)	
Primary sites of cancer			0.407
Lung n (%)	20 (90.9)	39 (83.0)	
Other sites n (%)	1 (4.5)	7 (14.9)	
Occult primary n (%)	1 (4.5)	1 (2.1)	
Cisplatin regimen			0.264
Single day n (%)	5 (22.7)	17 (36.2)	
Daily for 3 days n (%)	17 (77.3)	30 (63.8)	
Current chemotherapy cycle			0.006
Cycle 1 (%)	0 (0)	17 (36.2)	0.001
Cycle 2 (%)	5 (22.7)	11 (23.4)	0.950
≥Cycle 3 (%)	17 (77.3)	19 (40.4)	0.004
Chemotherapy purpose			0.678
First‐line n (%)	21 (95.5)	41(87.2)	
≥Second‐line n (%)	1 (4.5)	3 (6.4)	
Adjuvant chemotherapy n (%)	0 (0)	3 (6.4)	
Radiation history			0.705
Yes n (%)	1 (4.5)	5 (10.6)	
No n (%)	21 (95.5)	42 (89.4)	
Pretreatment ANC			0.391
≥2 × 10^9^/L (%)	22 (100)	43 (91.5)	
<2 × 10^9^/L (%)	0 (0)	4 (8.5)	

Abbreviations: ANC, absolute neutrophil count; CBCs, complete blood counts; PP, PEG‐rhG‐CSF prophylaxis.

### Comparison between baseline prechemotherapy CBC values and lowest mean EM CBC values post‐chemotherapy according to covariance analysis

There was no statistically significant difference in the baseline platelet counts between the PP‐Group (180.18 × 10^9^/L, 95% confidence interval [CI]: 136.76–223.60 × 10^9^/L) and the No‐PP‐Group (216.51 × 10^9^/L, 95% CI: 193.45–239.57 × 10^9^/L) (*p* = 0.103). After accounting for the baseline platelet levels by covariance analysis, no statistically significant difference was noted in the lowest EM post‐treatment platelet count between the PP‐Group (106.56 × 10^9^/L, 95% CI: 88.78–124.34 × 10^9^/L) and the No‐PP‐Group (127.70 × 10^9^/L, 95% CI: 115.62–139.78 × 10^9^/L) (*p* = 0.056). As for hemoglobin, the baseline mean for the PP‐Group was 111.05 g/L (95% CI: 102.95–119.14), significantly lower than that of the No‐PP‐Group, which was 126.00 g/L (95% CI: 121.77–130.23) (*p* < 0.01). After accounting for the baseline influence, there was no statistically significant difference in the lowest EM post‐treatment hemoglobin between the PP‐Group (110.48 g/L, 95% CI: 105.86–115.10 g/L) and the No‐PP‐Group (110.14 g/L, 95% CI: 107.08–113.19 g/L) (*p* = 0.906). The mean values of the baseline WBC were higher in the PP‐Group (8.05 × 10^9^/L, 95% CI: 6.53–9.57 × 10^9^/L) when compared with the No‐PP‐Group (6.67 × 10^9^/L, 95% CI: 5.84–7.50 × 10^9^/L), but the difference was not statistically significant (*p* = 0.082). The lowest EM means of the post‐treatment WBC in the PP‐Group were significantly higher than the values in the No‐PP‐Group, which were 4.95 × 10^9^/L (95% CI: 4.48–5.46 × 10^9^/L) and 2.80 × 10^9^/L (95% CI: 2.46–3.14 × 10^9^/L, respectively (*p* < 0.01), as indicated in Table 3.

**TABLE 3:** Pretreatment values of platelet, hemoglobin, and white blood cell, and the EM means of their post‐treatment counts at nadir between different PEG‐rhG‐CSF groups

	PP‐Group (*n* = 22)	No‐PP‐Group (*n* = 47)	*p*‐value
Platelet (10^9^/L)			
Pretreatment means (95% CI)	180.18 (136.76–223.60)	216.51 (193.45–239.57)	0.103
EM means for post‐treatment at nadir (95% CI)	106.56 (88.78–124.34)	127.70 (115.62–139.78)	0.056
Hemoglobin (g/L)			
Pretreatment means (95% CI)	111.05 (102.95–119.14)	126.00 (121.77–130.23)	<0.01
EM means for post‐treatment at nadir (95% CI)	110.48 (105.86–115.10)	110.14 (107.08–113.19)	0.906
White blood cell (10^9^/L)			
Pretreatment means (95% CI)	8.05 (6.53–9.57)	6.67 (5.84–7.50)	0.082
EM means for post‐treatment at nadir (95% CI)	4.95 (4.48–5.46)	2.80 (2.46–3.14)	<0.01

Abbreviations: CI, confidence interval; EM, estimated marginal; PP, PEG‐rhG‐CSF prophylaxis.

### Incidence of grade III or IV neutropenia

The incidence of grade III or IV neutropenia was significantly lower in the PP‐Group when compared with the No‐PP‐Group (9.1% vs. 68.1%, *p* < 0.01), as shown in Figure 3.

### Impact of the time interval between the administration of PEG‐rhG‐CSF and chemotherapy on safety

**FIGURE 2:**
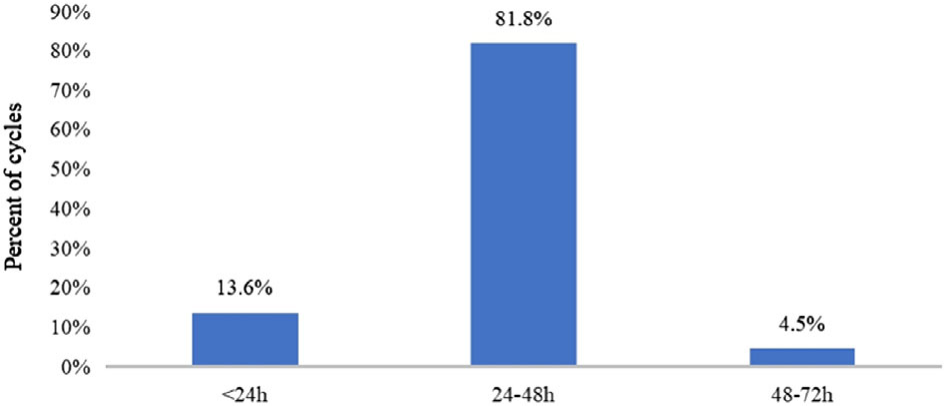
Prevalence of different intervals between PEG‐rhG‐CSF administration and completion of chemotherapy in the PP‐Group. PP, PEG‐rhG‐CSF prophylaxis

The interval between PEG‐rhG‐CSF administration and completion of chemotherapy of the PP‐Group is illustrated in Figure 2. A total of 13.6% (3/22) of cycles received PEG‐rhG‐CSF within less than 24 h after the last chemotherapy infusion. The minimum interval was 15 h. However, no grade II to IV myelosuppression and FN were observed in this subgroup.

**FIGURE 3:**
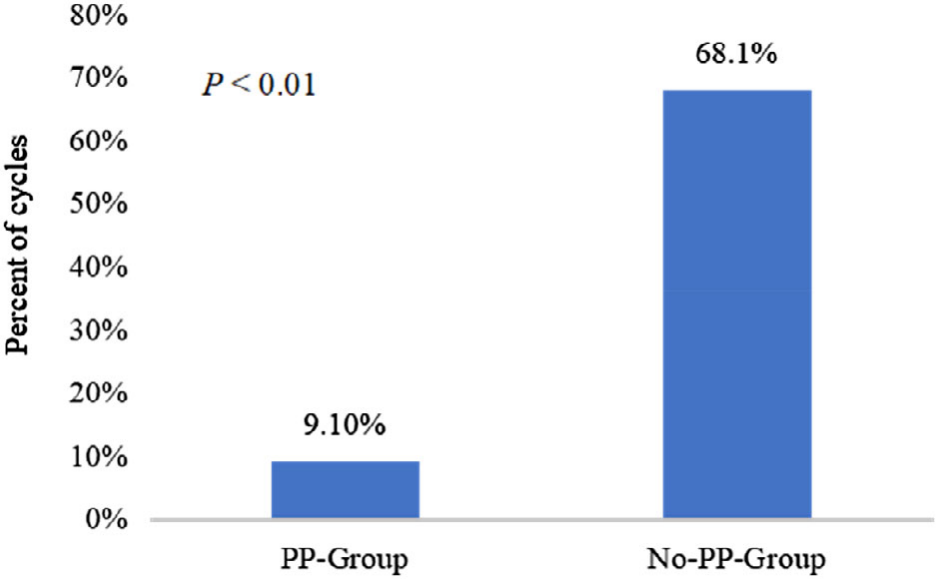
Incidence of grade III or IV neutropenia between the two groups. PP, PEG‐rhG‐CSF prophylaxis

## DISCUSSION

Patients receiving EP treatment for small cell carcinoma are at intermediate risk of developing FN. According to the NCCN guideline, prophylactic use of G‐CSF should be considered for patients in the intermediate‐risk group with one of the specific risk factors or for those who suffered FN or dose‐limiting neutropenic event in previous cycles. The administration of G‐CSF within 24 to 96 h after completion of chemotherapy is recommended by various professional bodies to minimize this risk.^11^, ^16^, ^17^ However, the optimal time interval between the cisplatin and PEG‐rhG‐CSF application is still controversial. Some physicians doubt whether it is safe to apply PEG‐rhG‐CSF only 24 h after EP regimen when there is still a certain amount of cisplatin present in the blood. Furthermore, the administration of half the standard 6 mg dose as it is currently being done in China may be as effective in reducing the risk of developing neutropenia. In our study, we, therefore, evaluated the impact of delivering a reduced dose (3 mg) of PEG‐rhG‐CSF within various time intervals following the EP regimen in reducing the risk of developing neutropenia.

Since cisplatin has a prolonged elimination half‐life value ranging from 30.5 to 106 h,^10^ the presence of cisplatin within the plasma may destroy any myelogenic activity produced by G‐CSF, eventually rendering the treatment ineffective. On the other hand, when evaluating the pharmacokinetics of cisplatin, one might argue that cisplatin tends to bind quickly with the blood protein, making it less toxic, while the toxic unbound cisplatin tends to be excreted within the first 2 h following infusion.^20^

In our study, 81.8% of the cycles in the PP‐Group received PEG‐rhG‐CSF within 24 to 48 h following the last chemotherapy dose. Irrespective of whether the cisplatin was delivered as a single dose on day one or daily for three days, a considerable amount of cisplatin was still present in the circulation when PEG‐rhG‐CSF was applied during that interval. However, in our study, we did not find an increased incidence of FN, antibiotic use due to neutropenia, or grade III or IV neutropenia in the PP‐Group when compared with the No‐PP‐Group. Furthermore, there was no significant difference in the platelet and hemoglobin counts post‐treatment between the groups. When compared with the No‐PP‐group, the WBC count in the PP‐group was significantly higher, while the incidence of grade III or IV neutropenia was significantly lower, indicating that the prophylactic treatment was effective in stimulating the production of neutrophils irrespective of the short time interval and reduced PEG‐rhG‐CSF dose. This finding supports the hypothesis that the concentration of the free platinum is low levels within the plasma, and hence the cisplatin will not interfere with the PEG‐rhG‐CSF, allowing for more flexibility in the interval between the two treatments and making it more convenient for the patient.^2^, ^6^, ^7^, ^8^, ^23^, ^24^, ^25^, ^26^

Various guidelines recommend at least a 24 h interval between PEG‐rhG‐CSF and chemotherapy.^11^, ^15^, ^21^ However, this time interval may still be too long and inconvenient for certain patients. In a cross‐sectional survey conducted in the United States, about 32% of patients received PEG‐rhG‐CSF within less than 24 h after completion of chemotherapy, and 43% of those patients received PEG‐rhG‐CSF on the same‐day across all chemotherapy cycles.^22^ In our study, there were also 13.6% of cycles in the PP‐Group who received PEG‐rhG‐CSF within less than 24 h after the cisplatin infusion (Figure 2). However, no severe myelosuppression and FN were observed throughout this treatment within this subgroup. These findings are in line with the updated American Society of Clinical Oncology (ASCO) recommendations that advise clinicians to still provide same‐day PEG‐rhG‐CSF if it is the only feasible means to administer prophylactic treatment for certain patients.^21^

An interesting finding of this study was that the baseline hemoglobin value of the PP‐Group was significantly lower than that of the No‐PP‐Group. This suggests a worse bone marrow reservation in the PP‐Group than in the No‐PP‐Group and confirms the rationality of our PEG‐rhG‐CSF prophylaxis in the real‐world setting. This was also consistent with the European Organization for Research and Treatment of Cancer (EORTC) guidelines published in 2010 that anemia is one of the risk factors for the development of severe neutropenia or FN.^27^ In addition, there was no statistically significant difference in the hemoglobin and platelet post‐treatment by covariance analysis. The administration interval had no negative influence on erythroid and megakaryocytic production.

From the pretreatment clinical characteristics, we found that most of the patients in the PP‐Group had a body weight of more than 60 kg, which meant they received PEG‐rhG‐CSFs at a dose of less than 100 μg/kg. However, irrespective of the patients' weight, findings indicate that the PP‐Group had a significantly lower incidence of grade III or IV neutropenia when compared with the No‐PP‐Group, which indicated that it was better than no prevention. These findings are consistent with a phase I clinical trial whereby 60 μg/kg and 100 μg/kg of PEG‐rhG‐CSF had similar effects in preventing neutropenia.^28^

No significant differences were found for the incidences of FN and antibiotic use related to neutropenia between the two groups, possibly as a result of the relatively small sample size in our study and the low incidences of these conditions in patients treated with EP regimes. The Chinese Society of Clinical Oncology (CSCO) guidelines suggests that the FN risk of EP regimen for small cell lung cancer is about 10% to 20%.^16^ The lower rate in our study might be related to the “WeChat” follow‐up group that allowed us to provide immediate advice when the patient reported symptoms of severe myelosuppression. The role of effective follow‐up procedures needs to be evaluated further in future studies.

The administration of a low dose (3 mg) of PEG‐rhG‐CSF within approximately 24 h or up to three days following EP treatment is safe and effective at reducing the risk of developing severe neutropenia. These findings suggest that the slowly metabolized cisplatin does not interfere with the PEG‐rhG‐CSF use, eventually allowing for a more flexible administration interval between the two treatments.

The study has several limitations that have to be acknowledged. Since this was a retrospective study, the CBCs were not always acquired at the same interval within the chemotherapy cycle, making it difficult to accurately quantify the time required to recover from severe myelosuppression, which is also an important index in judging the degree of bone marrow suppression. Additionally, PEG‐rhG‐CSF is usually administered using a fixed dose of 6 mg in many countries, but our study only covered the management of 3 mg. Therefore, the safety of larger doses warrants further investigation. In the absence of data of patients treated with the standard doses, we could only compare the efficacy of a 3 mg dose with no treatment. Further research is recommended to compare the efficacy of the 3 mg dose with the standard 6 mg dose. Finally, the sample size was too small and the data were collected from a single institution within a limited one‐year period, potentially limiting the generalizability of the research findings. Further randomized controlled studies using larger patient cohorts from multiple institutions to identify the optimal time intervals and dosage of PEG‐rhG‐CSF are therefore recommended.

## CONFLICT OF INTEREST

The authors declare that they have no conflict of interest associated with this study.
